# SHP-1 Promotes the Replication of Porcine Epidemic Diarrhea Virus by Inhibiting TRAF3-Mediated Type I Interferon Immune Responses

**DOI:** 10.3390/pathogens14101014

**Published:** 2025-10-07

**Authors:** Jiaqing Hu, Yuxin Kong, Yi Liu, Ning Li, Shijin Jiang

**Affiliations:** 1 Shandong Provincial Key Laboratory for Livestock Germplasm Innovation & Utilization, College of Animal Science and Technology, Shandong Agricultural University, No. 7 Panhe Street, Tai’an 271018, China; jqh0609@sdau.edu.cn; 2 Shandong Provincial Key Laboratory of Zoonoses, College of Veterinary Medicine, Shandong Agricultural University, No. 7 Panhe Street, Tai’an 271018, China; kyx1287017959@163.com (Y.K.); ly97651002@163.com (Y.L.)

**Keywords:** PEDV, SHP-1, IFN-β, TRAF3, ubiquitination

## Abstract

Porcine epidemic diarrhea virus (PEDV), a member of the genus *Alpha* coronavirus, is one of the main pathogens causing piglet diarrhea. PEDV can enhance its replication by regulating host protein function. The tyrosine phosphatase src homology 2 domain-containing PTP (SHP-1) acts as a host natural immune protein capable of influencing viral replication, but there are no studies on the regulation of virus replication by pig SHP-1. In this study, we expressed porcine SHP-1 protein and examined its interaction with PEDV as well as its potential role in PEDV infection. The results showed that SHP-1 overexpression in porcine kidney cells (PK15) significantly increased the mRNA level of viral S protein in a dose-dependent manner. In contrast, SHP-1 knockdown reduced S gene expression, indicating that SHP-1 promoted PEDV replication. Overexpression of SHP-1 had an inhibitory effect on *IFN-β*, *TNF-α*, *ISG15*, and *CXCL10*, while this inhibition was reduced as SHP-1 expression decreased. Furthermore, we found that SHP-1 interacted with TNF receptor-associated factor 3 (TRAF3) and inhibited its K63-linked ubiquitination, suppressing the expression of *IFN-β* and *ISG*s and facilitating PEDV replication. The study provided new insights for the prevention and control of porcine epidemic diarrhea.

## 1. Introduction

Porcine epidemic diarrhea (PED), caused by porcine epidemic diarrhea virus (PEDV), is an acute infectious disease. It is characterized by diarrhea, vomiting, anorexia, dehydration, and weight loss; the morbidity and mortality rates are very high in neonatal piglets [1]. PED was first reported in Britain [2] and Belgium [3] in the late 1970s. The classic strain CV777 was successfully isolated in Belgium for the first time in 1978 [3]. This isolate was subsequently developed into a live-attenuated vaccine for the control of PED. However, the new PEDV variant strain emerged in China in 2010 [4] and changed the prevalence map of this disease. The epidemiological data showed that the variant PEDV strain was prevalent in 29 provinces in China during 2011–2014, and rates of PEDV-positive pig farms were 71.43–83.47% [5]. The piglets were extremely sensitive to the variant strain, with a mortality rate of 80% to 100% [6]. The reason was that the high mutated rates scattered in the neutralizing epitope domain of the variant strain could help the virus to escape the recognition of host immune cells [7,8]. Due to the absence of effective vaccines, this disease is currently causing serious damage to the pig industry.

The innate immunity serves as the first line of defense against invading pathogens and is characterized by increased type I interferon (IFN) production. Generally, the innate immune response is triggered by the activation of pattern recognition receptors (PRRs), such as toll-like receptors (TLRs) and retinoic acid inducible gene-I (RIG-I)-like receptors (RLRs) [9]. Upon recognition of viral or bacterial components, known as pathogen-associated molecular patterns (PAMPs), PRRs activate intracellular signaling cascades by interacting with adaptor proteins. This process leads to the translocation and phosphorylation of transcription factors NF-κB, IFN regulatory factor (IRF) 3, and IRF7, which induce the production of type I IFN. Specifically, mitochondrial antiviral signaling protein (MAVS) acts as an adaptor for RLR signaling. Upon RLR activation, MAVS ubiquitinates tumor necrosis factor receptor-associated factor 3 (TRAF3). Activated TRAF3 recruits tank-binding kinase 1 (TBK1), IKKε, and NF-κB essential modulator (NEMO) to form a complex, which phosphorylates and dimerizes IRF3/IRF7. The phosphorylated dimers translocate to the nucleus, bind interferon-stimulated response elements (ISREs), and drive type I IFN and IFN-stimulated gene (ISG) expression. In addition, MAVS can also recruit TRAF6-IKKα-IKKβ-NEMO complex, and then NF-κB is activated and enters the nucleus, inducing the production of type I IFN and inflammatory cytokines [10,11]. The signal transduction pathway mediated by TIR domain-containing adapter-inducing interferon-β (TRIF), an adaptor protein of TLRs, is similar to MAVS, which also plays an important role in antiviral immune responses [12].

It is noteworthy that the precise regulation of innate immune responses relies on the involvement of key signaling molecules. Among them, the tyrosine phosphatase protein SHP-1, an important negative regulatory factor, plays a critical role in maintaining immune homeostasis. SHP-1 contains two random N-terminal Src homology (SH) 2 regions (N-SH2 and C-SH2), a classical catalytic protein tyrosine phosphatase (PTP) domain, and a C-terminal tail region containing multiple phosphorylation sites [13]. SHP-1 is highly expressed in blood-derived cells and epithelial cells [14]. SHP-1 negatively regulates signaling pathways by binding phosphorylated molecules (e.g., receptor tyrosine kinases, immune receptor-associated molecules) and dephosphorylating them to terminate/attenuate signal transduction. The overexpression of SHP-1 facilitated the replication of herpes simplex virus type 1 (HSV-1) and vesicular stomatitis virus (VSV) in L929 cells by inhibiting K63-linked ubiquitination of TRAF3 and reducing the production of type I IFN and ISGs [15]. Additionally, SHP-1 maintains cellular homeostasis by inhibiting excessive proliferation, abnormal activation, or defective apoptosis [16,17].

PEDV has evolved many strategies to impair the production of IFN to escape host innate immunity. Previous studies have shown that PEDV non-structural protein (nsp) 14 and nsp1 inhibited type I IFN by degrading RIG-I and CREB-binding protein, respectively [18,19]. PEDV nucleocapsid protein circumvented host antiviral immunity by disrupting the TBK1-IRF3 interaction [20]. While SHP-1 is recognized as an important negative regulator of immune responses, its role in porcine antiviral innate immunity remains poorly characterized. It is necessary to clarify whether PEDV modulates type I IFN responses through SHP-1. Therefore, in this study, the effect of porcine SHP-1 on PEDV replication was studied. It was found that SHP-1 overexpression promoted the reproduction of PEDV, and then the underlying mechanism of this result was investigated. These findings provide a basis for further revealing the mechanism of porcine SHP-1 in innate immunity and new ideas for scientific prevention and control of PED.

## 2. Materials and Methods

### 2.1. Cells and Viruses

Vero cells, porcine kidney cells (PK15), and human embryonic kidney 293T cells (HEK-293T) were purchased from the ATCC resource center. The accession number of the Vero cell is CCL-81, PK15 is CCL-33, and HEK-293T is CRL-3216. The cells were preserved in our laboratory and cultured in Dulbecco’s modified Eagle medium (DMEM) (Hyclone, Logan, UT, USA) supplemented with 10% (*v*/*v*) heat-inactivated fetal bovine serum (Sigma, St Louis, MO, USA) and 100 U/mL penicillin and streptomycin (Hyclone, Logan, UT, USA). The classical PEDV strain CV777 was preserved in our laboratory and propagated in Vero cells. Sendai virus (SeV) was presented by Prof. Yingli Shang (College of veterinary medicine, Shandong Agricultural University, Tai’an City, China) and was propagated in specific pathogen-free (SPF) chicken embryos.

### 2.2. Porcine SHP-1 Gene Clone and Plasmid Constructions

The total RNA of PK15 cells was extracted using TRIzol reagent (Takara, Dalian, China) following the manufacturer’s instructions. The concentrations of all samples were measured using an ultraviolet spectrophotometer (Shimadzu, Shimazu, Japan), and cDNA was synthesized from 1 μg total RNA using ReverTra AceR qPCR RT Master Mix with the gDNA Remover kit (Toyobo Co., Ltd., Osaka, Japan). Then, it was used as a template for amplifying target genes. The genes coding RIG-I signaling proteins, including *RIG-I*, *MAVS*, *TRAF3*, *TBK1*, *IRF3*, *IRF7*, and *SHP-1*, were amplified by PCR using the primers (Table 1), and the Hieff Clone^®^ Universal One Step Cloning Kit (YEASEN, Shanghai, China) was used to construct the recombinant expression plasmids, namely, pCAGGS-HA-SHP-1 (pHA-SHP-1), pEGFP-SHP-1, pCAGGS-FLAG-RIG-1 (pFLAG-RIG-I), pFLAG-MAVS, pFLAG-TRAF3, pFLAG-TBK1, pFLAG-IRF3, and pFLAG-IRF7. Positive plasmids were confirmed by sequencing (Tsingke Ltd., Qingdao, China).

### 2.3. Quantitative Real-Time PCR (qPCR)

The template cDNA for qPCR was obtained according to the methods described above. qPCR was performed on Roche LightCycler 96 (Roche, Basel, Switzerland) using the TB GreenTM Fast qPCR Mix (Takara, Dalian, China). The reaction volume was 20 µL, including 10 µL 2 × TB Green Premix Ex Taq II (Takara, Dalian, China), 1 µL template cDNA, 0.8 µL of each forward and reverse primer (10 µM), and 7.4 µL sterile water. The qPCR conditions were as follows: one cycle at 95 °C for 30 s, followed by 40 cycles at 95 °C for 5 s and 60 °C for 30 s. The melting curve was analyzed to confirm specific amplification. Each sample was analyzed three times.

### 2.4. The Effect of Porcine SHP-1 on PEDV Replication

PK15 cells were cultured in 6-well plates and grown overnight to approximately 80% confluence. The cells were transfected with pEGFP-SHP-1 plasmid (2 µg, 3 µg, and 4 µg) using Lipfectanine 2000 (Thermo Fisher Scientific, Waltham, MA, USA), with pEGFP empty vector serving as the control. Eight hours post-transfection, cells were infected with PEDV (MOI = 5), and cells were collected at 16 h post-infection (hpi) for measuring mRNA expressions of the PEDV S gene by the qPCR method (Table 2).

To further verify the effect of endogenous SHP-1 on PEDV replication, three pairs of siRNAs were designed against porcine SHP-1 protein: si894, si1511, si276, and a negative control siRNA. They were synthesized by Sango Biotech (Sango Biotech, Shanghai, China) and were transfected into PK15 cells (10 pM/well). The interference effect was detected at 36 h and 48 h by qPCR. The selected siRNA (si1511) was transfected into PK15 cells for 20 h. Then, the cells were infected with PEDV at an MOI of 5, the total cellular RNA was extracted at 16 hpi, and the changes of PEDV replication were detected by qPCR as described above.

### 2.5. The Effects of Porcine SHP-1 on the Expression of IFN-β and Cytokines

The different doses of pHA-SHP-1 plasmids (2 µg, 3 µg, and 4 µg) were transfected into PK15 cells in 6-well plates, respectively. The cells were inoculated with 1 MOI of SeV at 8 h post-transfection, and the expressions of *IFN-β*, *TNF-α*, *ISG15*, and *CXCL10* were detected by qPCR (Table 2). Similarly, PK15 cells were transfected with si1511 (10 pM) and then infected with SeV (MOI = 1) to further analyze the effect of porcine SHP-1 on the *IFN-β* and cytokines.

### 2.6. Dual Luciferase Reporter Assay

The dual luciferase reporter gene plasmid pRL-TK, porcine pIFN-β-Luc, and pISRE-Luc promoter reporter plasmids were kept in our laboratory. HEK-293T cells were cultured in 24-well plates and transiently co-transfected with the pIFN-β-Luc (100 ng/well), pRL-TK (50 ng/well), pHA-SHP-1 (300 ng/well), and pFLAG-RIG-I (200 ng/well) plasmids. Similarly, pIFN-β-Luc, pRL-TK, pHA-SHP-1, and other plasmids involved in the RLR signal pathway, including pFLAG-MAVS, pFLAG-TRAF3, pFLAG-TBK1, and pFLAG-IRF3, were co-transfected, respectively, and the empty vector served as a negative control. The cell lysate samples were collected 24 h after transfection for luciferase activity detection using a dual luciferase reporter assay system (Beyotime, Shanghai, China).

### 2.7. Immunofluorescence Assay

Prior to cell seeding, sterile glass coverslips were placed in 24-well culture plates to prepare slides for subsequent analysis. The prepared slides were co-transfected with pFlag-TRAF3, pEGFP-SHP-1, and pEGFP empty plasmid, respectively. Twenty-four hours post-transfection, cells were fixed with 4% (*w*/*v*) paraformaldehyde at 4 °C for 20 min. Cells were rinsed with sterile PBS three times for 5 min each and then permeabilized with 0.1% TritonX-100 (Sigma, Darmstadt, Germany) for 10 min. The cells were blocked with 5% skimmed milk powder for 1 h, incubated with mouse anti-flag monoclonal antibody (ABclonal, Beijing, China) (Code: AE005) for 1.5 h at room temperature, and then washed with PBS 3 times. Then, the cy3-labeled goat anti-mouse antibody (Beyotime, A0521) was added and incubated for 1 h at room temperature. After washing, nuclei were stained with 2-(4-amidinophenyl)-6-indolecarboxamidine dihydrochloride (DAPI) for 30 min at 37 °C. Immunofluorescence was visualized with a Leica SPE confocal microscope (Leica, Wetzlar, Germany).

### 2.8. Immunoprecipitation and Western Blot Analysis

HEK-293T cells in 6-well plates were co-transfected with pFlag-TRAF3 and pHA-SHP-1 plasmids and further cultured for 24 h. The cells were harvested and lysed with cold RIPA lysis buffer (Beyotime, Shanghai, China). The supernatant of the whole-cell lysate was collected after centrifugation at 12,000× rpm for 10 min and incubated overnight at 4 °C with magnetic bead-conjugated mouse anti-flag mAb (ABclonal, AE037). The supernatant was removed, and the magnetic beads were washed 5 times with NP40 lysate containing a PMSF protease inhibitor (G-Clone, Beijing, China). The immunoprecipitated protein complexes were detected by protein immunoblotting with mouse anti-flag and mouse anti-HA antibodies, respectively (Abclone, Beijing, China). The precipitates and lysates were boiled in 1× sodium dodecyl sulfate (SDS) loading buffer for 10 min at 100 °C. Proteins were separated by 12% gel electrophoresis and then transferred onto polyvinylidene difluoride membranes (PVDF, Millipore, Bedford, MA, USA) using a semi-dry transfer device according to standard procedures. Subsequently, the membranes were blocked with 5% skim milk for 2 h at room temperature. After three 5 min washes with TBST, the membrane was probed with primary antibodies, including mouse anti-flag (ABclonal, AE005), mouse anti-HA (ABclonal, AE008), rabbit anti-GFP (ABclonal, AE078), and mouse anti-β-actin (ABclonal, AC004) overnight at 4 °C. The membranes were incubated for 1 h at room temperature with HRP-conjugated secondary antibodies: goat anti-mouse IgG (H + L) (Beyotime, A0350) and goat anti-rabbit IgG (Beyotime, A0208) after washing three times with TBST. Proteins were visualized by enhanced chemiluminescence according to the manufacturer’s instructions. The protein bands were visualized using an ECL system (Bio-Rad, Shanghai, China), and band intensities were analyzed using ImageJ/Fiji software (NIH, Bethesda, MD, USA).

### 2.9. Statistical Analysis

All results were obtained from three repeated biological experiments. The results were expressed as the means ± SD, and Student’s *t*-test was used to analyze the data. Statistical analyses and data visualization were performed using GraphPad Prism 9 (GraphPad, La Jolla, CA, USA). Statistical significance is represented by an asterisk, *, and *p* < 0.05, which is a significant difference. ** and *p* < 0.01 indicate a highly significant difference.

## 3. Results

### 3.1. Overexpression of Porcine SHP-1 Promotes Replication of PEDV

**Figure 1:**
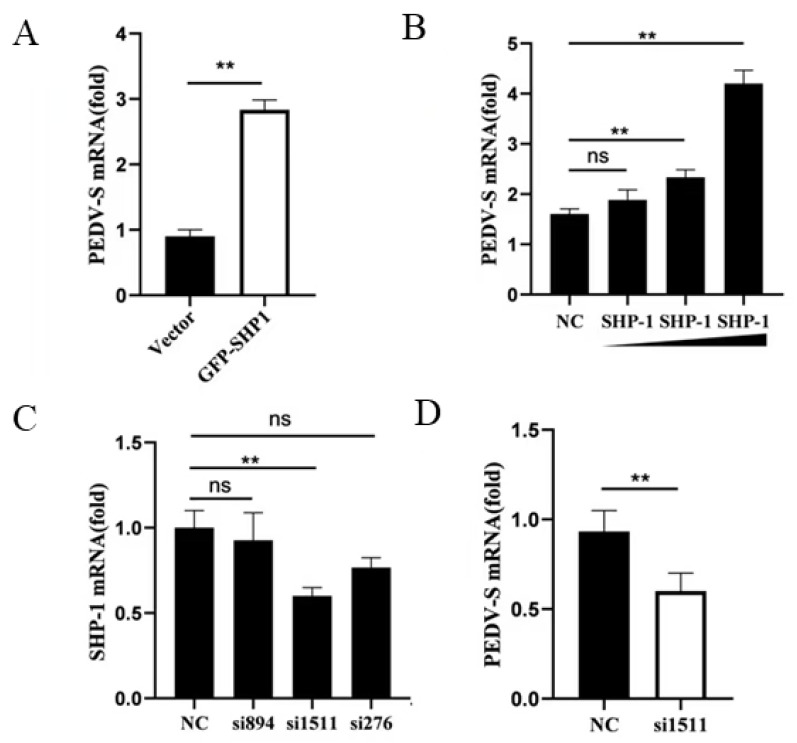
SHP-1 enhanced the replication of PEDV. (**A**) The pEGFP-SHP-1 plasmid (3 µg) was transferred into PK15 cells. After 8 h, the cells were infected with PEDV (MOI = 5) and cultured for 16 h, and the viral S gene was detected by qPCR. (**B**) The different doses of SHP-1 plasmids (2 µg, 3 µg, and 4 µg) were transfected into PK15, the cells were infected with PEDV (MOI = 5), and the S gene was detected. (**C**) Three pairs of siRNAs against porcine SHP-1 and Negatine Control (NC) siRNA were transfected into PK15 cells, and the interference effect was assessed by detecting the mRNA. (**D**) si1511 was transfected into PK15 cells for 20 h, the cells were infected with PEDV at 5 MOI, and the changes of the viral S gene were detected by qPCR. The results were expressed as the means ± SD (*n* = 3). ** *p* < 0.01.

To investigate the role of porcine SHP-1 in PEDV replication, PK15 cells were infected with PEDV after pEGFP-SHP-1 plasmid transfection, and mRNA expression of the PEDV S gene was detected using qPCR. The result showed that the transcript levels of S protein in the experimental group increased and were significantly higher than those in the control group (Figure 1A), indicating that SHP-1 overexpression significantly enhanced PEDV replication. Moreover, PEDV replication gradually increased with growing transfection doses (Figure 1B).

To further analyze the effect of SHP-1 on PEDV replication, siRNAs against SHP-1 were synthesized and transfected into PK15 cells. The knockdown effects were first detected, and si1511 could significantly reduce SHP-1 transcription expression (Figure 1C). The relative expression of the PEDV S gene was detected in the cells transfected with si1511; the results demonstrated that inhibition of SHP-1 protein could significantly reduce the expression of the S gene (Figure 1D).

### 3.2. Porcine SHP-1 Downregulates Transcription of IFN-β and Several Cytokines

**Figure 2:**
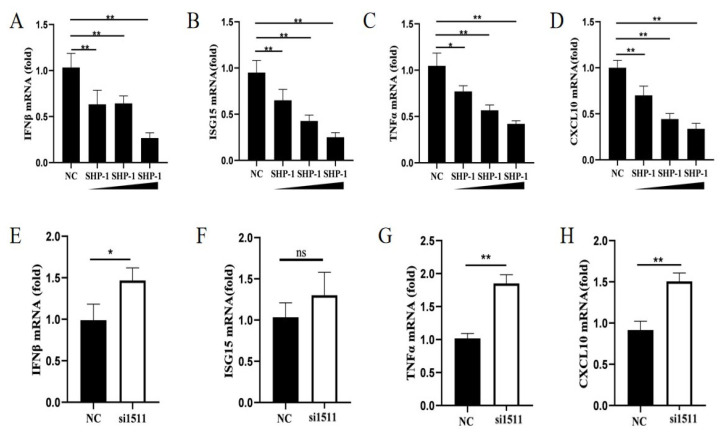
SHP-1 downregulated transcription levels of some cytokines. pCAGGS-HA-SHP-1 plasmids (2 µg, 3 µg, and 4 µg) were transferred into PK15 cells, and cells were inoculated with SeV (MOI = 1) 8 h post-transfection. The mRNA relative expressions of *IFN-β* (**A**), *ISG15* (**B**), *TNF-α* (**C**), and *CXCL10* (**D**) were measured by qPCR. si1511 was transfected into PK15 cells to analyze the effect of SHP-1 knockdown on the mRNA levels of *IFN-β* (**E**), *ISG15* (**F**), *TNF-α* (**G**), and *CXCL10* (**H**). The results were expressed as the means ± SD (*n* = 3). * *p* < 0.05, ** *p* < 0.01.

Mouse SHP-1 served as a negative regulator of antiviral immune responses and inhibited the production of type I IFN and pro-inflammatory cytokines induced by virus infection [16]. To explore the effect of porcine SHP-1 on type I IFN and cytokines, different doses of SHP-1 were transfected into PK15 cells, followed by SeV infection, and qPCR was performed to detect the expression of *IFN-β*, *ISG15*, *CXCL10*, and *TNF-α*. The results showed that SHP-1 overexpression significantly inhibited *IFN-β* (Figure 2A). Similarly, the expression of *ISG15*, *TNF-α*, and *CXCL10* was also inhibited, with a dose dependence (Figure 2B–D).

We further analyzed the effect of endogenous SHP-1 on *IFN-β*, *ISG15*, *CXCL10*, and *TNF-α*. The results demonstrated that SHP-1 knockdown significantly promoted the expression of *IFN-β*, *TNF-α*, and *CXCL10* (Figure 2E,G,H). Although there was no significance, the transcription of *ISG15* also increased (Figure 2F). These results indicated that porcine SHP-1 may regulate the type I IFN signaling pathway to influence the replication of PEDV.

### 3.3. Porcine SHP-1 Protein Targets TRAF3 to Negatively Regulate Type I IFN

**Figure 3:**
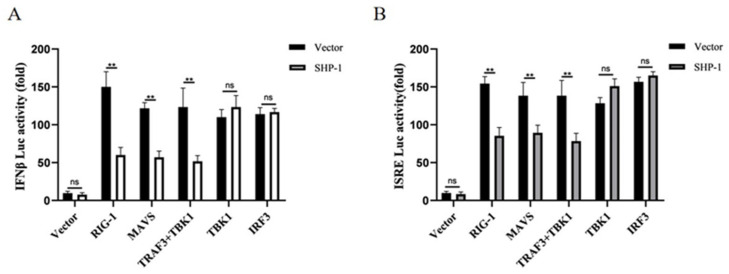
SHP-1 antagonized IFN-β response mediated by the RIG-I signaling pathway. SHP-1 and the other plasmids (RIG-I, MAVS, TRAF3 + TBK1, TBK1, IRF3) were co-transfected into HEK-293T cells, and the effects of SHP-1 on IFN-β (**A**) and ISRE promoters (**B**) were analyzed. The results were expressed as the means ± SD (*n* = 3). ** *p* < 0.01.

The RLR signaling pathway plays a crucial role in the initiation of type I IFN responses. To identify how porcine SHP-1 suppressed the expression of IFN-β, we analyzed the effect of porcine SHP-1 on RLR-mediated IFN-β production using the dual luciferase reporter assay. As shown in Figure 3A, porcine SHP-1 significantly inhibited the IFN-β induced by RIG-I and MAVS proteins, but it had no effect on the activation of TBK1 and IRF3, which were both localized downstream of TRAF3. The same results were also detected in the expression of ISRE (Figure 3B). The results indicated that TRAF3 may be a target protein of porcine SHP-1.

### 3.4. Porcine SHP-1 Interacted with TRAF3 and Inhibited K63 Ubiquitination

**Figure 4:**
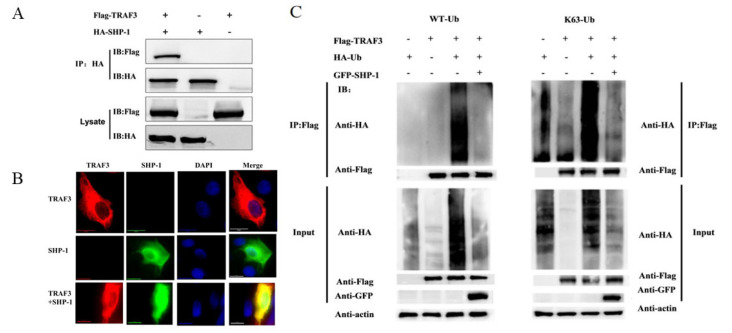
Interaction between porcine SHP-1 and TRAF3. (**A**) The pHA-SHP-1 and pFlag-TRAF3 plasmids were co-transfected into HEK-293T cells for 24 h. The immunoprecipitation test was conducted to analyze the interaction between SHP-1 and TRAF3. (**B**) pEGFP-SHP-1 and pFlag-TRAF3 were co-transfected into PK15 cells, and the confocal test was performed. (**C**) In the presence and absence of pGFP-SHP-1, HEK-293T cells were co-transfected with plasmids pFlag-TRAF3 and wild-type ubiquitination (WT-Ub) or K63-Ub. After 24 h, the cell lysate was harvested, and the immunoprecipitation test was performed to analyze the effect of SHP-1 on TRAF3 ubiquitination.

To determine the interaction between porcine SHP-1 and TRAF3, co-immunoprecipitation and indirect immunofluorescence assays were conducted in cells. The results showed that porcine SHP-1 interacted with TRAF3 protein (Figure 4A), and both exhibited significant co-localization in PK15 cells (Figure 4B).

Ubiquitination of TRAF3 is particularly important in inducing the production of type I IFN. To analyze whether SHP-1 inhibited TRAF3 ubiquitination and the type of ubiquitination, the recombiant plasmids TRAF3, SHP-1, and HA-Ub were transfected into HEK-293T cells. As shown in Figure 4C, the ubiquitination of TRAF3 was obvious in the co-transfected group of TRAF3 and WT-Ub, but the overexpression of SHP-1 inhibited the TRAF3 ubiquitination, and further tests showed that SHP-1 could inhibit the K63-linked ubiquitination of TRAF3.

## 4. Discussion

PED has caused great economic losses in the global swine industry due to its high mortality. While previous studies showed that PEDV effectively suppressed IFN production, recent research suggested that multiple host proteins were also involved in the regulation of the RLR signaling pathway during PEDV infection, promoting or inhibiting IFN production. For instance, autophagy-associated protein (ATG4B) and trans-reactive DNA-binding protein (TARDBP) can inhibit PEDV replication by promoting the expression of interferon [21]. Conversely, the heteronuclear protein U degraded TRAF3 at the transcription level to inhibit IFN-β and facilitate virus replication [22].

SHP-1 plays a crucial role in negatively regulating the immune responses. A deficiency in SHP-1 resulted in a loss of immune self-limiting in the body, leading to excessive release of cytokines and chemokines. The massive secretion of cytokines and chemokines triggered a severe inflammatory response, which caused organ damage and reduced the body’s disease resistance. However, PEDV-induced chemokines CXCL-9 and CXCL-13 attracted T cells and B cells, respectively [23]. The expression of pro-inflammatory cytokines and chemokines can also exert an anti-PEDV effect. Yu et al. reported that overexpression of IL-1α, IL-1β, TNF-α, CCL2, CCL5, and CXCL8 could significantly inhibit PEDV replication in Vero E6 cells [24], and intramuscular immunization with CCL25 and CCL28 adjuvants inactivated PEDV and elicited substantial protection against a virulent PEDV challenge in 5-week-old pigs [25]. In terms of the effect on viral replication, it was reported that SHP-1 participated in regulating virus replication in cells and effectively inhibited the IFN production induced by HSV-1, VSV, etc. [17]. Similar results were observed in fish immunization; SHP-1 overexpression was able to reduce the induction of IFN by VSV, and ISGs expression was also significantly reduced [26]. SeV potently activates the host innate immune response, as exemplified by the induction of type I IFN and ISGs. Consequently, it is widely utilized in studies investigating immune signaling pathways and antiviral immune responses [27]. In the current study, SHP-1 was transfected into PK15 cells, followed by SeV infection, and we found that porcine SHP-1 promoted PEDV replication by inhibiting the production of type I IFN and some cytokines. But, unfortunately, the porcine-derived cells lacking SHP-1 were not constructed to further study the effect of this host protein on PEDV replication, which was a limitation of this study. Additionally, SHP-1 may play different roles in different innate immune signaling pathways. It has been shown that homeobox A10 (HoxA10) interacts with p38 mitogen-activated protein kinase (MAPK) and recruits SHP-1 to inhibit HBV replication [28].

The RLR signaling pathway is critical for exerting antiviral responses. SHP-1 acts on key proteins in the RLR pathway; it can block this signaling pathway and inhibit production [16]. Therefore, in order to determine whether porcine SHP-1 could inhibit RLR-mediated type I IFN expression, we performed a dual luciferase reporter assay. The results showed that porcine SHP-1 significantly inhibited the activation of IFN-β and ISRE promoters by RIG-I and MAVS but could not inhibit TBK1- and IRF3-induced IFN production, which was located downstream of TRAF3 protein, indicating that SHP-1 may participate in an inhibitory role at a point upstream of TBK1-IRF3. Further experiments revealed that both porcine SHP-1 and TRAF3 co-localized in PK15 cells, and SHP-1 interacted with TRAF3 protein. TRAF3 is an important host protein involved in several innate immune signal transduction pathways, including the RLR signaling pathway. In the RLR signaling pathway, activated MAVS undergoes dimerization and recruits E3 ligase or its own ubiquitinated TRAF3 to its TIM region, forming a complex of TRAF3, TBK1, IKKε, etc., finally inducing IFN expression [27]. Thus, ubiquitination of TRAF3 is a key event in the eventual induction of interferon production. Multiple ubiquitination pathways exist in the organism, such as K6, K11, K27, K29, K33, K48, and K63, among which K63 and K48 ubiquitination are the two main ubiquitination pathways. K48 ubiquitination is mainly responsible for protein degradation, while K63 ubiquitination is mainly related to the activation of multiple signaling pathways [29]. To clarify the mechanism of action of porcine SHP-1 in inhibiting type I IFN, cells were co-transfected with SHP-1 and TRAF3, followed by an ubiquitination assay, and it was found that SHP-1 significantly inhibited the total ubiquitination of TRAF3. Further, after co-transfection of the K63 ubiquitination plasmid and SHP-1, TRAF3 displayed consistent results as expected. These results demonstrated that porcine SHP-1 exerts its IFN inhibitory effect by inhibiting the K63-linked ubiquitination of TRAF3 in the RLR signaling pathway. This mechanism aligned with previous reports of murine SHP-1 targeting TRAF3 to reduce IFN production [15]. Nevertheless, the molecular mechanism underlying the interaction between porcine-specific SHP-1 and TRAF3 during PEDV infection, as well as its functional divergence from murine SHP-1, requires further investigation.

## 5. Conclusions

In summary, this study confirmed that overexpression of porcine SHP-1 can inhibit IFN-β and promote the replication of PEDV. Mechanically, porcine SHP-1 exerted an inhibitory effect by targeting TRAF3 and inhibiting its K63-linked ubiquitination in the RLR signaling pathway. This study further enhanced the knowledge of the role of porcine SHP-1 in antiviral immunity.

## Figures and Tables

**Table 1:** Amplification primers of target genes involved in the RLR signaling pathway.

Genes	Primer Sequences (5′-3′)
*RIG-I*	F: gacgataaggaattcgagctcATGACAGCAGAGCAGCGGC
	R: ccgggtaccatcgatgagctcTCACTCAAGGTTGCCCATTCC
*MAVS*	F: gacgataaggaattcgagctcATGACGTTTGCCGAGGACAA
	R: ccgggtaccatcgatgagctcTCACTGGGGCAGGCGCCG
*TRAF3*	F: gatgacgacgataaggaattcATGACACACAGAATGGAGCCG
	R; attaagatctgctagctcgagTCAGGGGTCAGGCAGATCC
*TBK1*	F: gacgataaggaattcgagctcATGCAGAGCACTTCTAATCATCTTTG
	R: ccgggtaccatcgatgagctcCTAAAGACAGTCAACATTGCGAAGG
*IRF3*	F: gacgataaggaattcgagctcATGGGAACTCAGAAGCCTCGG
	R: ccgggtaccatcgatgagctcCTAGAAATCCATGTCCTCCACCA
*IRF7*	F: gacgataaggaattcgagctcATGGCCGCGGCTCCTGAC
	R: ccgggtaccatcgatgagctcCTAGGCCGGCTGCTCCAC
*SHP-1*	F: gttccagattacgctgaattcATGTTGTCCCGTGGGTGGT
	R: attaagatctgctagctcgagTCACTTCCTCTTCAGGGAGCC

**Table 2:** The primer sequences of qPCR used in this study.

Genes	Primer Sequences (5′-3′)
*qSHP-1*	F: GCAGATGGTGTGGCAGGAGAAC
	R: GTGACAGCGTAGGGTCCGTAAAC
*qIFN-β*	F: TGCATCCTCCAAATCGCTCT
	R: ATTGAGGAGTCCCAGGCAAC
*qTNF-α*	F: ACCACGCTCTTCTGCCTACTG
	R: TGAGACGATGATCTGAGTCCTTGG
*qISG15*	F: GGCAGCACAGTCCTGTTGATGG
	R: TGCGTCAGCCAGACCTCATAGG
*qCXCL10*	F: ACTGTTCGCTGTACCTGCATCAAG
	R: GCCTTCGACTCTGGATTCAGACATC
*qPEDV-S*	F: ACTGCCTATCCAACAAAGCCATTCC
	R: TTGTCGCAACACGGGACCAATC

## Data Availability

The data supporting this study’s findings are available upon request from the first author (J.H.).
